# The Role of Transperineal Ultrasound for the Assessment of the Anorectal Angle and Its Relationship with Levator Ani Muscle Avulsion

**DOI:** 10.3390/tomography8030105

**Published:** 2022-05-06

**Authors:** José Antonio García-Mejido, Sara García-Pombo, Cristina Fernández-Conde, Carlota Borrero, Ana Fernández-Palacín, José Antonio Sainz-Bueno

**Affiliations:** 1 Department of Obstetrics and Gynecology, Valme University Hospital, 41014 Seville, Spain; sgarciapombo@gmail.com (S.G.-P.); cristinafconde@hotmail.com (C.F.-C.); carlota_borrero@hotmail.com (C.B.); jsainz@us.es (J.A.S.-B.); 2 Department of Obstetrics and Gynecology, University of Seville, 41014 Seville, Spain; 3 Biostatistics Unit, Department of Preventive Medicine and Public Health, University of Seville, 41014 Seville, Spain

**Keywords:** anorectal angle, levator ani muscle, avulsion, ultrasound, transperitoneal ultrasound, pelvic floor

## Abstract

The relationship between the anorectal angle (ARA) and the levator ani muscle (LAM) is well known. In this study, we aimed to demonstrate that the ARA changes when LAM avulsion occurs after vaginal delivery. This was a secondary, observational retrospective study with data obtained from three previous studies. Using transperineal ultrasound, the presence of avulsion was assessed when abnormal insertion of the LAM was observed in three central slices. In addition, the ARA was assessed in the midsagittal plane (at rest, in Valsalva and at maximum contraction) as the angle between the posterior border of the distal part of the rectum and the central axis of the anal canal. The ARA was higher in patients with bilateral LAM avulsion than in patients without LAM avulsion at rest (131.8 ± 14.1 vs. 136.2 ± 13.8), in Valsalva (129.4 ± 15.5 vs. 136.5 ± 14.4) and at maximum contraction (125.7 ± 15.5 vs. 132.3 ± 13.2). The differences between both groups expressed as the odds ratio (OR) adjusted for maternal age were 1.031 (95% confidence interval (CI), 1.001–1.061; *p* = 0.041) at rest, 1.036 (95% CI, 1.008–1.064; *p* = 0.012) in Valsalva and 1.031 (95% CI, 1.003–1.059; *p* = 0.027) at maximum contraction. In conclusion, LAM avulsion produces an increase in the ARA at rest, during contraction and in Valsalva, especially in cases of bilateral LAM avulsion.

## 1. Introduction

**Figure 1:**
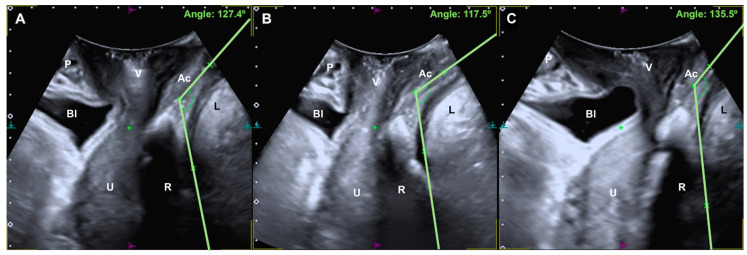
A, Anorectal angle at rest (**A**), with maximum contraction (**B**) and with the Valsalva maneuver (**C**). P, pubic bone; Bl, bladder; V, vagina; U, uterus; R, rectal ampulla; Ac, anal canal; L, levator ani muscle.

The mechanism of anal continence is complicated and depends on the integrity of different components, such as innervation, sphincter function (internal and external anal sphincter), the anorectal angle (ARA), rectal distensibility, fecal consistency, and intestinal motility. The coordinated function of all of these components allows the maintenance of anal continence and voluntary defecation. In fact, the ARA is coordinated with sphincter relaxation, with the angle increasing to facilitate voluntary defecation [1]. The ARA is the angle demarcating the junction between the rectum and the anal canal, which is produced by the puborectalis muscle (a component of the levator ani) to form a sling around this level, creating a soft posterior rectal impression demarcating this anorectal junction [2] (Figure 1). The ARA during defecation is wide (a more obtuse angle) to facilitate feces evacuation, allowing emptying of the rectum without significant descent of the pelvic floor [2]. After defecation, the puborectalis returns to its contracted state (a more acute angle), the anal canal closes to restore the ARA, and the pelvic floor returns to its normal resting state [2].

The damage produced during childbirth in the components that influence the mechanisms of anal continence are well known, as also observed in high-grade sphincter tears. However, sphincter lesions are not the only alterations that can affect anal continence after vaginal delivery. A reduced curvature has been described in the anorectal junction during voluntary activation of the puborectal sling after delivery of the first child [3]. In addition, an increase in the ARA in the immediate puerperium that recovers over time has been reported, with an increase in the ARA during the performance of Valsalva [4]. On the other hand, childbirth is also the main risk factor for levator ani muscle (LAM) avulsion, which is present in 10–35% of women after a vaginal delivery [5,6], with instrumental vaginal delivery being the main risk factor [6]. The most critical moment for the appearance of LAM avulsion is when the vertex of the fetal head is at the +3 or +4 station [7], since this is when the area of the levator hiatus reaches its greatest size [8].

The relationship between the ARA and the LAM is well known since the ARA is formed by the sling that forms the LAM in the posterior part of the rectum demarcating the anorectal junction. Logically, this angle may depend on the integrity of the insertion of the LAM at the level of the pubis, with LAM avulsion being a factor that may influence expansion of the ARA. Therefore, the objective of our work is to demonstrate that the ARA changes when LAM avulsion occurs after vaginal delivery.

## 2. Materials and Methods

### 2.1. Subjects

This is a secondary, retrospective observational study with data obtained from three previous studies [9,10,11]. Patients were recruited between September 2012 and September 2019. The studies were approved by Andalucia’s Board of Biomedicine Ethics Committee under codes 0545-N-18 [9], 0153-N-17 [10] and 3004/2012 [11]. In these studies, LAM avulsion during operative vaginal delivery [10,11] and the changes that occur after physical therapy in patients with LAM avulsion [9] were studied.

The inclusion criteria were as follows: women who were primiparous, had a vaginal delivery in the cephalic position (normal or instrumental), were at term gestation (37–42 weeks), had no prior pelvic floor corrective surgery, and provided written informed consent were considered eligible for the study and were therefore included. Subjects with dyssynergia, anismus, fecal incontinence, or anal prolapse were not included.

### 2.2. Data Collection

The obstetric parameters evaluated were maternal age in years, gestational age in weeks, labor induction, epidural analgesia, epidural onset to delivery in minutes, the duration of second stage of labor in minutes, episiotomy and perineal tears according to Sultan’s classification of perineal tears [12]. The fetal parameters studied after birth were fetal weight in grams and head circumference in centimeters.

### 2.3. Ultrasound Assessment

Ultrasound imaging was performed six months after delivery by a single examiner with specific training in 3D pelvic floor ultrasound (JAGM). Prior to and throughout the ultrasound assessment, the examiner was blinded to obstetric data relating to the delivery and clinical manifestations. A 500^®^ Toshiba Aplio (Toshiba Medical Systems Corp., Tokyo, Japan) ultrasound with a PVT-675MV 3D abdominal probe was used.

The mode of acquisition and offline analysis of volumes have been described in previous studies [13]; the volumes were acquired in the midsagittal plane of the pelvic floor with the woman in the lithotomy position after voiding. Three volume measurements were taken for each patient: at rest, with the Valsalva maneuver, and with maximum contraction. Avulsion was defined based on maximum contraction in the multislice mode described above [14,15]. Complete avulsion was diagnosed when abnormal insertion of the LAM (LAM detachment) was observed in three central slices. In unclear cases, a levator–urethra gap > 2.5 cm was used to define abnormal insertion [16].

For the assessment of the ARA, the convex probe was placed transperineally in a longitudinal manner, and the anorectal canal, the anorectal junction and the rectal ampulla were visualized. The ARA was measured in the midsagittal plane (at rest, in Valsalva and at maximum contraction) and was defined as the angle between the posterior border of the distal part of the rectum and the central axis of the anal canal [17,18,19,20,21,22]. This ARA measurement method is similar to that performed in previous studies using defecography and magnetic resonance [17,18] (Figure 1).

### 2.4. Statistical Analysis

Quantitative variables are presented as the means and standard deviations, and qualitative variables are presented as percentages. For quantitative variables, the normality of the data was contrasted (Shapiro–Wilk test) in the groups defined by the type of delivery, and Student’s t test for independent samples or the nonparametric Mann–Whitney U test was applied according to whether normality was verified. For qualitative variables, either contingency tables and chi-square tests or non-asymptotic Monte Carlo methods and exact tests were applied. We used univariate binary logistic regression analysis to determine crude odds ratios and multivariate binary logistic regression analysis to control for possible confounding factors. *p* < 0.05 was considered statistically significant.

To detect a difference of 10 degrees in the anorectal angle at rest between the groups with and without avulsion considering a common standard deviation of 13 degrees (from a preliminary study), an α error of 5% and an β error of 10% (power of 90%), we needed 41 women in each group.

## 3. Results

The volumes of 260 patients studied in previous studies could be extracted; 188 did not have LAM avulsion, and 72 had LAM avulsion (41 unilateral and 31 bilateral). The general data of the patients included according to the presence or absence of LAM avulsion are shown in Table 1. Age was the only parameter that showed statistically significant differences among patients without avulsion, patients with unilateral LAM, and patients with bilateral LAM (29.0 ± 5.7 vs. 29.8 ± 5.2 vs. 32.8 ± 4.3; *p* = 0.001).

Table 2 compares the ARA between patients without LAM avulsion and patients with unilateral LAM avulsion. We observed no statistically significant differences in the measurements of the ARA at rest, in Valsalva and during maximum contraction between the groups.

The differences in the measurements of the ARA at rest, in Valsalva and at maximum contraction between patients without LAM avulsion and patients with bilateral LAM avulsion are shown in Table 3. The ARA was greater in patients with LAM avulsion than in patients without LAM avulsion at rest (131.8 ± 14.1 vs. 136.2 ± 13.8), in Valsalva (129.4 ± 15.5 vs. 136.5 ± 14.4), and at maximum contraction (125.7 ± 15.5 vs. 132.3 ± 13.2). The differences between both groups expressed as the OR adjusted for maternal age were 1.031 at rest (95% CI, 1.001–1.061; *p* = 0.041), 1.036 (95% CI, 1.008–1.064; *p* = 0.012) in Valsalva and 1.031 at maximum contraction (95% CI, 1.003–1.059; *p* = 0.027).

Table 4 shows the variations in the measurements of the ARA at rest, in Valsalva and at maximum contraction between patients with unilateral LAM avulsion and patients with bilateral LAM avulsion. The ARA was higher in patients with bilateral LAM avulsion than in patients with unilateral LAM avulsion at rest (129.4 ± 12.5 vs. 136.2 ± 13.8), in Valsalva (127.9 ± 13.5 vs. 136.5 ± 14.4) and at maximum contraction (130.2 ± 13.5 vs. 132.3 ± 13.2). The differences were statistically significant when studying the ARA at rest and in Valsalva, and the ORs adjusted for maternal age were 1.044 (95% CI, 1.003–1.088; *p* = 0.037) and 1.052 (95% CI, 1.010–1.096; *p* = 0.014), respectively.

## 4. Discussion

We observed that patients with bilateral LAM avulsion had a higher ARA than those without LAM avulsion at rest (131.8 ± 14.1 vs. 136.2 ± 13.8), in Valsalva (129.4 ± 15.5 vs. 136.5 ± 14.4) and at maximum contraction (125.7 ± 15.5 vs. 132.3 ± 13.2). In addition, when comparing the ARAs of patients with bilateral LAM to those of patients with unilateral LAM, we found that the angle continues to be greater in cases of bilateral LAM avulsion at rest (129.4 ± 12.5 vs. 136.2 ± 13.8), in Valsalva (127.9 ± 13.5 vs. 136.5 ± 14.4) and at maximum contraction (130.2 ± 13.5 vs. 132.3 ± 13.2). No differences were observed between the different groups. Therefore, the variations in the ARA between the different groups were determined by the presence of LAM avulsion. The normal ARA has been established to range from 94 to 114 degrees at rest and to change between 15 and 20 degrees among rest, contraction or defecation [23]. We found higher mean ARA values that may be explained by the increase in the ARA during puerperium, as has been previously described [24]. In fact, in studies using two-dimensional transperineal ultrasound, a decrease in anorectal mobility has been observed three months after delivery [3], and the levator sling excursion decreased [3], suggesting that the ARA is closely related to MEA. Therefore, logically, LAM avulsion causes an increase in the ARA by not maintaining the same posterior rectal impression that occurs when the LAM is normally inserted, which is consistent with the data that we extracted from our study. In fact, the ARA widens more when LAM avulsion is bilateral, since the LAM does not have any muscular insertion at the level of the pubis to continue maintaining a more acute ARA, as occurs in patients with an intact LAM or with unilateral LAM avulsion.

The value of the ARA remains controversial, with multiple attempts to standardize the modifications that the ARA undergoes in the course of a woman’s life. However, the ARA is relevant in the late development of mild pathology, such as dyssynergia or anismus, and more severe conditions such as fecal incontinence or anal prolapse [25]. The International Continence Society established that a smaller resting ARA may suggest an increase in tone in the MEA, producing a reduction in the ARA during contraction and widening during Valsalva [26]. However, in the puerperium, a functional deficit has been reported in the pelvic floor muscles, followed by spontaneous and partial recovery six months after delivery [27]. Therefore, ARA recovery has been proposed to be delayed to 6 and 12 months after delivery [3]. From our data, we observed that during the Valsalva maneuver, the mean ARAs were either not increased or were reduced in the three study groups, which may be justified by the possible dyssynergia that may be present in some cases at six months after delivery.

The main strengths of this paper are that it addresses an interesting and very timely question and provides a clear answer, with a sufficient number of cases included to support the objectives of the study. The influence of LAM avulsion on the ARA was established, which may indicate that in the future, the LAM should be studied in clinical cases of anal incontinence. The imaging technique used (transperineal ultrasound) for the study of the ARA may be a limitation, since most studies that define the ARA are performed with defecography or MRI [17,18,19,20,21,22]. Nevertheless, ultrasound has been previously shown to be comparable to defecography for the study of the ARA [28]. Currently, the intraobserver and interobserver variability for ARA measurement by transperitoneal ultrasound remains undescribed, which presents an opportunity for possible future publications.

## 5. Conclusions

In conclusion, LAM avulsion produces an increase in the ARA at rest, at maximum contraction and in Valsalva, especially in cases of bilateral LAM avulsion.

## Figures and Tables

**Table 1:** General characteristics of the patients included according to the presence or absence of LAM avulsion.

	Without Avulsion (*n* = 188)	Avulsion		P Global	P ^a^	P ^b^	P ^c^
		Unilateral (*n* = 41)	Bilateral (*n* = 31)				
Maternal age in years, mean (SD)	29.0 ± 5.7	29.8 ± 5.2	32.8 ± 4.3	0.001	1	0.001	0.048
Gestational age in weeks, mean (SD)	39.6 ± 1.1	39.6 ± 1.3	39.6 ± 1.4	0.736	---	---	---
Induction of labor, *n* (%)	41 (21.8%)	8 (19.5%)	6 (19.4%)	0.916	---	---	---
Epidural anesthesia, *n* (%)	179 (95.2%)	40 (97.6%)	29 (93.5%)	0.808	---	---	---
Epidural onset to delivery in minutes, mean (SD)	388.3 ± 194.6	413.7 ± 260.5	410.3 ± 166.6	0.785	---	---	---
Second stage of labor in minutes, mean (SD)	100.5 ± 62.7	122.1 ± 88.5	85.1 ± 52.0	0.123	---	---	---
Episiotomy, *n* (%)	145 (77.1%)	37 (90.2%)	28 (90.3%)	0.055	---	---	---
Perineal tear, *n* (%)	89 (47.3%)	18 (43.9%)	19 (61.3%)	0.290	---	---	---
Grade I	31 (34.8%)	7 (38.9%)	4 (21.1%)	0.699	---	---	---
Grade II	48 (53.9%)	10 (55.6%)	12 (63.2%)				
Grade III	10 (11.2%)	1 (5.6%)	3 (15.8%)				
Grade IV	0 (0%)	0 (0%)	0 (0%)				
Birth weight in grams, mean (SD)	3321.7 ± 370.2	3447.4 ± 399.5	3433.7 ± 419.7	0.098	---	---	---
Fetal head circumference in cm, mean (SD)	34.4 ± 1.2	34.8 ± 1.4	35.2 ± 4.1	0.423	---	---	---

P ^a^ = comparison between the without avulsion group and unilateral avulsion group. P ^b^ = comparison between the without avulsion group and unilateral avulsion group. P ^c^ = comparison between the unilateral avulsion group and bilateral avulsion group.

**Table 2:** The ARA in patients without LAM avulsion and in patients with unilateral LAM avulsion. ORs were adjusted for maternal age in years.

	Without Avulsion (*n* = 188)	Unilateral Avulsion (*n* = 41)	*p*	Crude OR	95% CI	Adjusted P	Adjusted OR	Adjusted 95% CI
Anorectal angle (°)								
At rest, mean (SD)	131.8 ± 14.1	129.4 ± 12.5	0.330	0.988	0.964–1.012	0.364	0.989	0.965–1.013
In Valsalva, mean (SD)	129.4 ± 15.5	127.9 ± 13.5	0.587	0.994	0.972–1.016	0.627	0.995	0.973–1.017
At maximum contraction, mean (SD)	125.7 ± 15.5	130.2 ± 13.5	0.090	1.020	0.997–1.043	0.085	1.020	0.997–1.044

**Table 3:** The ARA in patients without LAM avulsion and in patients with bilateral LAM avulsion. ORs were adjusted for maternal age in years.

	Without Avulsion (*n* = 188)	Bilateral Avulsion (*n* = 41)	*p*	Crude OR	95% CI	Adjusted P	Adjusted OR	Adjusted 95% CI
Anorectal angle (°)								
At rest, mean (SD)	131.8 ± 14.1	136.2 ± 13.8	0.107	1.023	0.995–1.052	0.041	1.031	1.001–1.061
In Valsalva, mean (SD)	129.4 ± 15.5	136.5 ± 14.4	0.018	1.033	1.006–1.062	0.012	1.036	1.008–1.064
At maximum contraction, mean (SD)	125.7 ± 15.5	132.3 ± 13.2	0.028	1.030	1.003–1.057	0.027	1.031	1.003–1.059

**Table 4:** The ARA in patients with unilateral LAM avulsion and in patients with bilateral LAM avulsion. ORs were adjusted for maternal age in years.

	Unilateral Avulsion (*n* = 41)	Bilateral Avulsion (*n* = 31)	*p*	Crude OR	95% CI	Adjusted P	Adjusted OR	Adjusted 95% CI
Anorectal angle (°)								
Rest, mean (SD)	129.4 ± 12.5	136.2 ± 13.8	0.040	1.042	1.002–1.084	0.037	1.044	1.003–1.088
Valsalva, mean (SD)	127.9 ± 13.5	136.5 ± 14.4	0.016	1.048	1.009–1.089	0.014	1.052	1.010–1.096
Maximum contraction, mean (SD)	130.2 ± 13.5	132.3 ± 13.2	0.501	1.012	0.977–1.049	0.503	1.013	0.976–1.051

## Data Availability

The data are kept by the main author.
